# Effect of Nk-lysin peptides on bacterial growth, MIC, antimicrobial resistance, and viral activities

**DOI:** 10.1080/10495398.2023.2290520

**Published:** 2023-12-15

**Authors:** Haitham A. Yacoub, Maged Mostafa Mahmoud, Ahmed M. Al-Hejin, Turki S. Abujamel, Shams Tabrez, Sherif Abd-Elmaksoud

**Affiliations:** a Cell Biology Department, Biotechnology Research Institute, National Research Centre, Cairo, Egypt; b Regerenative Medicine Unit, King Fahd Medical Research Center, King Abdulaziz University, Jeddah, Saudi Arabia (SA); c Department of Medical Laboratory Sciences, Faculty of Applied Medical Sciences, King Abdulaziz University, Jeddah, Saudi Arabia (SA); d Biological Sciences Department, Faculty of Sciences, King Abdulaziz University, Jeddah, Saudi Arabia; e Vaccines and Immunotherapy Unit, King Fahd Medical Research Center, King Abdulaziz University, Jeddah, Saudi Arabia; f Environmental Virology Laboratory, Water Pollution Research Department, Environmental Research Institute, National Research Centre, Cairo, Egypt

**Keywords:** Beta-lactamase genes, bovine, chicken, human, Nk-lysin, *rotavirus*

## Abstract

NK-lysins from chicken, bovine and human are used as antiviral and antibacterial agents. Gram-negative and gram-positive microorganisms, including *Streptococcus pyogenes, Streptococcus mutans, Escherichia coli, Pseudomonas aeruginosa, Klebsiella oxytoca, Shigella sonnei, Klebsiella pneumoniae* and *Salmonella typhimurium*, are susceptible to NK-lysin treatment. The presence of dominant TEM-1 gene was noted in all untreated and treated bacteria, while TOHO-1 gene was absent in all bacteria. Importantly, β-lactamase genes CTX-M-1, CTX-M-8, and CTX-M-9 genes were detected in untreated bacterial strains; however, none of these were found in any bacterial strains following treatment with NK-lysin peptides. NK-lysin peptides are also used to test for inhibition of infectivity, which ranged from 50 to 90% depending on NK-lysin species. Chicken, bo vine and human NK-lysin peptides are demonstrated herein to have antibacterial activity and antiviral activity against Rotavirus (strain SA-11). On the basis of the comparison between these peptides, potent antiviral activity of bovine NK-lysin against Rotavirus (strain SA-11) is particularly evident, inhibiting infection by up to 90%. However, growth was also significantly inhibited by chicken and human NK-lysin peptides, restricted by 80 and 50%, respectively. This study provided a novel treatment using NK-lysin peptides to inhibit expression of β-lactamase genes in β-lactam antibiotic-resistant bacterial infections.

## Introduction

The antimicrobial peptides (AMPs) are immune-response molecules found in various organisms. AMPs serve a critical function in the initial line of defense against invading pathogens by inhibiting characteristics that mediate fitness to induce infection. Furthermore, AMPs have immune-modulatory properties.^1^^,^^2^ Traditional medicinal chemistry, increased immune activation, specialized vaccinations, probiotics, modified bacteriophages, and adjuvants are now being used to identify new antibacterial therapeutics.^3–5^ Until May 2019, 407 preclinical studies focused on antibacterial medicines created using a wide range of techniques, including ''non-traditional’' tactics (not small molecule pharmaceuticals and/or directly acting on target bacteria.^6^^,^^7^ diverse AMPs have been isolated throughout all kingdoms of diverse species since their discovery, from bacteria to insects, plants and mammals.^8^ The vast distribution and diversity of AMPs imply a variety of activities, including immunomodulation, bactericidal activity and synergism between distinct AMPs.^9–11^ NK-lysin is a cationic AMP with antibacterial action that was discovered in the gastrointestinal tract of pigs. It was also shown to be released from the granules of natural killer and cytotoxic T cells, giving rise to the term NK-lysin. In humans, NK-lysin is referred to as granulysin and is a member of the saposin-like protein family. The peptide has a significant positive charge due to its conserved cysteine residues, which are also important for the development of disulfide bonds inside the amino acid chain. The 3-D structural properties have been found to boost its antibacterial potential.^12–14^

The NK-lysin peptide’s capacity to pass across bacterial membranes is significantly dependent on the quantity of non-polar amino acids on its side chain. As a result, these non-polar amino acids are highly conserved and account for 27–51% of the amino acid residues in all animal species’ NK-lysin homologues. Non-polar amino acids like Ile and Leu enhance peptide binding and cell membrane rupture. NK-lysin and granulysin peptides share a saposin domain with modest differences in domain length. The domain is folded in 4–5 helical bundles depending on the species, with three disulfide linkages between six cysteine residues that stabilize the peptide conformational shape.^15^

Antibiotic resistance is a dynamic issue, with fresh facts and thoughts constantly altering and redefining the area. The most interesting issues in this discipline are -lactam antibiotic resistance and, more specifically, β-lactamases, which are enzymes capable of hydrolyzing β-lactam antibiotics.^16^ Since the 1980s, the number of identified β-lactamases has increased significantly; however, the healthcare and pharmaceutical industries have considered alternative methods to improve and invent new routes to combat and prevent multi-drug resistance pathogens by using biocompatible nontoxic agents.

Recently, there have been several attempts to find reliable inhibitors of -lactamase enzymes.^17^^,^^18^ The principal process for addressing -lactamase-mediated resistance is the combination of susceptible -lactams with mechanisms based on β-lactamase inhibitors.^19^ Several studies have shown that innate immune response molecules can have antiviral effects on enveloped and non-enveloped viruses, and that membrane disruption is an approach of action used to combat viral infection.^20–22^ The NK-lysin short peptide of the turbot (*Scophthalmus maximus*) recently demonstrated potent antiviral activity against spring viremia of carp virus (SVCV), preventing viral particles from binding to host cells and also preventing virus-cell membrane integration, which requires a low pH.

Although research on the antibacterial potential of NK-lysin peptides has been carried out, there has been no study on the antiviral activity of bovine, human and chicken NK-lysin peptides against *Rotavirus* has been conducted. *Rotaviruses* are the primary cause of acute gastroenteritis in babies and young children, accounting for an estimated 215,000 fatalities globally each year.^23^ These viruses are non-enveloped and contain a genome made up of 11 double-stranded RNA segments.^24^ Infection with Rotavirus in adults is often asymptomatic or moderate, although individuals may be severely immunocompromised.^25^ Rotaviruses are shed in the feces of both sick children and adults, leading the virus to spread fast among the population.^26^ The purpose of this study was to investigate the antibacterial and antiviral activity of NK-lysin peptides from chickens, bovine and humans, as well as the mechanism of action.

## Materials and methods

The study protocol was approved by the Ethics Committee of King Abdulaziz University (Reference No 325-19). This study was conducted based on the health and ethical guidelines and informed consent obtained from the participants.

### Peptides

The synthesis of (30 amino acid) of mature peptides of bovine, human and chicken NK-lysin peptides was carried out by GenScript (USA Inc.). HPLC was used to purify the three peptides up to 95%, and mass spectrometry analysis showed the peptides mass less than 1 Dalton of the theoretical value (Table 1). The kanamycin was obtained from Sigma Aldrich (St Louis, MO, USA). The antibiotic was dissolved in sterile water or 0.9% (*w/v*) NaCl and kept at −20 °C to make a stock solution. This solution was used within 2 weeks of its creation. The determined concentrations showed the number of active antibiotics in micrograms per unit volume (µg/ml).

**Table 1.** The amino acids sequence of the three Nk-lysin peptides.

Peptide name	Amino acid sequence	M.W (Dalton)
Chicken-Nk-lysin	PDEDAINNALNKVCSTGRRQRSICKQLLKK	3399.92
Bovine- Nk-lysin	RPSKNVIIHVTSNVCSKMGLWSILCNQMMK	3419.16
Human-Nk-lysin	PTQRSVSNAATRVCRTGRSRWRDVCRNFMR	3568.07

### Bacterial cell preparation

The two Gram-positive bacterial strains utilized in this study: *Streptococcus pyogenes* (ATCC 19615) and *Streptococcus mutans* were obtained from the patient at King Abdulaziz University Hospital, Jeddah, Saudi Arabia. The other five species of Gram-negative strains used in this study were: *Escherichia coli* (ATCC 11775), *Pseudomonas aeruginosa* (ATCC 9027), *Klebsiella oxytoca* (ATCC 49131), *Shigella sonnei* (ATCC 25931) and *Salmonella typhimurium* (ATCC 14028). Tryptic soy broth was used to grow all bacterial species overnight, after which the cells were washed using phosphate-buffered saline (PBS; 8 g/L NaCl, 0.2 g/L KCl, 1.4 g/L Na_2_HPO_4_ and 0.24 g/L KH_2_PO_4_) and then diluted within the same buffer (consisting of 1/1000 Tryptic Soy Broth (TSB)) to an OD 600 nm (0.08–0.1, 1 × 10^5^) colony-forming units (CFU)/ml determined after performing retrospective plate counts on the TS agar. Most of the experiments employed this inoculum preparation, apart when minimum inhibitory and bactericidal concentrations were being examined, we employed cells in Muller–Hinton broth (MHB).

### Antimicrobial activity assays

#### Minimum inhibitory and bactericidal concentrations

Under the Clinical Laboratory and Standard Institute (CLSI) broth micro-dilution technique as presented by,^27^ the smallest inhibitory and highest bactericidal concentrations of the NK-lysin of bovine, chicken and human peptides were estimated in the tested species. Here, a Muller–Hinton broth comprising of 0.01% *v/v* acetic acid (Sigma Aldrich, St Louis, MO, USA) and 0.2% *w/v* bovine serum albumin (Sigma Aldrich) was used. The aerobic incubation of bacterial cells diluted to 0.5 × 10^5^ CFU ml^−1^ in MHB was carried out on a microplate using an equivalent volume (50 µl) of varying concentrations of the NK-lysin peptides of bovines, humans and chickens, which were in the range of 0–150 µg/ml and the plate was incubated at 37 °C. For each well, the optical densities were noted at 0 minutes; 1, 2 and 3 hours; and overnight. MIC values were used to denote the smallest peptide concentrations that decreased the growth of the tested microorganisms to 50%.

### Colony count assays

The testing of the antimicrobial functions of the NK-lysin of bovine, human and chicken peptides was carried out against *Salmonella typhimurium* (ATCC 14028). The bacteria were kept in a Tryptic soy broth at 37 °C and were grown to the mid-logarithmic stage prior to testing. The bacteria cells were pelleted and then re-suspended in a 10 mM sodium phosphate buffer at a pH 7.0 after being diluted to OD600 of 0.08 (bacterial culture) within MHB. Then, 50 µl of the NK-lysin of chicken, human and bovine peptides were combined with the equal volume (50 µl) of bacterial culture, after which they were incubated at room temperature for 3 hours. The cultures (1 µl) were then diluted 1000 times and spread over Muller–Hinton (MH) agar plates. The colonies were counted after 24 hours at room temperature to determine the number of survived bacteria.

### Growth kinetic activity

The growth kinetic abilities of the NK-lysin of bovine, human and chicken peptides were gauged as per the method described in.^27^ The aerobic incubation of bacterial cells diluted to 0.5 × 10^8^ CFU ml^−1^ in MHB was carried out on a microplate with the same volume (50 µl) of 2× MIC concentrations of the NK-lysin peptides of chickens, humans and bovines. The plate was then incubated at room temperature. The optical densities were noted at 0 minutes, 1, 2, 3 and 4 hours for each well. A triplicate well was used to obtain the kinetic potential of NK-lysin peptides thrice.

### Bacterial lytic potential

The protocol of Falco et al.^28^ was used to perform the assay for the screening of the bactericidal potential of the Nk-lysin of human and bovine peptides using two different bacterial inoculums [O.D 600 (0.5) and O.D 600 (1.0)]. In the bacterial culture, the Nk-lysin peptides (1× MIC) were added in the same volume. The microplate was subsequently incubated at room temperature. BioTek Instruments, Inc. reader was used to measure the micro-plates at varied time intervals.^29^ The findings were presented as a ratio of O.D 600 nm at each time interval compared to the OD 600 nm at 0 minutes (as a percentage).

### Mode of action of NK-lysin peptides

#### Leakage of intracellular contents

To determine the loss of DNA/RNA^30^ protocol was used with some changes. Briefly, 50 µl of bacteria was combined with the same volume of NK-lysin of bovine, human and chicken peptides at their 2× MIC and then incubated at room temperature. Sample collection was carried out at different time intervals (15, 30 and 60 minutes), which were diluted (1:10) and then filtered across 0.22 µm pores (Merck, Tullagreen, Ireland). The measurement at O.D 260 nm of the filtrates was carried out over Novus NanoDrop plate (Greiner Bio-one GmbH, Frickenhausen, Germany). The results were presented as a ratio to the original OD 260 nm.

### Detection of *beta-lactamase* enzymes and quinolone resistance genes

#### Bacterial DNA extraction

The two Gram-positive bacterial strains utilized in this study: *Streptococcus pyogenes* (ATCC 19615) and *Streptococcus mutans* were obtained from the patient at King Abdulaziz University Hospital, Jeddah, Saudi Arabia. The other five species of Gram-negative strains used in this study were: *Escherichia coli* (ATCC 11775), *Pseudomonas aeruginosa* (ATCC 9027), *Klebsiella oxytoca* (ATCC 49131), Shigella *sonnei* (ATCC 25931) and *Salmonella typhimurium* (ATCC 14028). Luria Bertani (LB) agar was used to grow the bacterial strains at room temperature overnight. Inoculation of a single colony was performed in 5 ml LB broth, which was grown in a shaking incubator at room temperature for 16–18 hours. After this, genomic DNA was obtained using QIAGEN genomic DNA extraction kit (QIAGEN, USA).

### Characterization of *beta-lactamase* enzymes

PCR was performed to amplify the target genes to detect and characterize the beta-lactamase (bla) along with certain oligonucleotide primers (Table 2). The PCR conditions include an initial denaturation at 94 °C for 5 min followed by 35 cycles; each for 30s at 94 °C, specific annealing temperature for 30s and extension for 30s at 72 °C followed by final extension for 10 min at 72 °C.^31^ Once ethidium bromide staining was achieved, the PCR amplicons were inserted in 1.5% gel electrophoresis and visualized using gel documentation system.

**Table 2.** The primers list for detection and characterization of beta-lactamases and quinolone resistance genes.

Gene	Forward primer (5′ to 3′)	Reverse primer (5′ to 3′)	Amplicon size (bp)	References
BLATem	ATGAGTATTCAACATTTCCG	GACAGTTACCAATGCTTAATCA	869	Hendriksen et al. [71]
CTX-M1	GACGATGTCACTGGCTGAGC	AGCCGCCGACGCTAATACA	499	Hendriksen et al. [71]
TOHO1	GCGACCTGGTTAACTACAATCC	CGGTAGTATTGCCCTTAAGCC	351	Hendriksen et al. [71]
CTXM8	CGCTTT GCCATGTGCAGCACC	GCT CAGTACGATCGAGCC	307	Hendriksen et al. [71]
CTXM9	GCTGGAGAAAAGCAGCGGAG	GTAAGCTGACGCAACGTCTG	474	Hendriksen et al. [71]
Gyrase A	AAATCTGCCCGTGTCGTTGGT	GCCATACCTACTGCGATACC	344	Harrois et al. [72]
QnrA	ATTTCTCACGCCAGGATTTG	GATCGGCAAAGGTTAGGTCA	516	Piddock [69]
QnrB	GATCGTGAAAGCCAGAAAGG	ACGATGCCTGGTAGTTGTCC	469	Piddock [69]
QnrD	CGAGATCAATTTACGGGGAATA	AACAAGCTGAAGCGCCTG	565	Kruger et al. [73]
QnrS	ACGACATTCGTCAACTGCAA	TAAATTGGCACCCTGTAGGC	417	Piddock [69]

### Characterization of quinolone resistance genes

The genomic DNA was examined for presence of plasmid mediated quinolone resistance genes and quinolone resistance determining region (QRDR) using specific primer sets (Table 2). The PCR conditions include an initial denaturation at 94 °C for 5 min followed by 35 cycles; each for 30s at 94 °C, specific annealing temperature for 30s and extension for 30s at 72 °C followed by final extension for 10 min at 72 °C.^31^ Once ethidium bromide staining was achieved, the PCR amplicons were inserted in 1.5% gel electrophoresis and then visualized using gel documentation system.

### Antiviral activity of Nk-lysin peptides

#### Cytotoxicity experiments

The Nk-lysin of chicken, bovine and human peptides cytotoxicity was examined according to^32^ after making 80 µg/ml stock concentration using sterile deionized H_2_O. Briefly, 2-fold serial dilutions were carried out of the dissolved peptides and MA-104 cell monolayers were used to inoculate 100 µL of each dilution. An inverted light microscope was utilized to examine cell morphology daily for 6 days.

### Rotavirus and cell culture preparation

Rotavirus (strain SA-11) was activated by 10 µg/mL trypsin before propagating on MA-104 cells derived from African rhesus monkey kidney cells. Cells were cultivated in tissue culture flasks encompassing less than 5% CO_2_ placed at 37 °C in Dulbecco’s Minimal Essential Medium (DMEM; Sigma-Aldrich Co., St. Louis, MO, USA) along with an additional 1% antibiotic antimycotic solution and 10% heat inactivated fetal bovine serum (Sigma-Aldrich Co., St. Louis, MO, USA). Centrifugation of rotavirus was performed at 300 ×g for 5 mins in order to remove cell debris and make them pure. The supernatant was utilized as a stock suspension after filtration through 0.2 µm membrane. The stock virus contained 10^6^–10^7^ TCID50/mL and were stored at −80 °C for future use.

### Antiviral efficacy

100 μl Of the Nk-lysin peptides was mixed with 100 μl of activated rotavirus SA-11 (1 × 10^6^ TCID50/ml). The mixture was incubated at 37 °C for only 1 hour. After that, the cells were placed into 96-well tissue culture plates (Nunclon, Roskilde, Denmark) and incubated until density reached approximately 5.0 × 10^4^ cells per well. After that, the cells were infected with 100 μl of 10-fold dilutions of treated (virus + tested peptide) and untreated rotavirus SA-11 followed by an incubation period encompassing 5% CO_2_ for 5 days at 37 °C. In order to activate infectivity, the rotavirus was placed in trypsin solution of concentration 10 µg/mL at 37 °C for 30 min. The cytopathic effect (CPE) was observed after microscopic monitoring of the cells. The viral concentration was represented as the infectious dose per milliliter of tissue culture (TCID50/mL), equal to 50 percent of wells showing CPE at a specific dilution of the sample. The tissue culture infectious does that affected TCID50 were determined using titration results obtained by the Reed–Muench method (Payment and Trudel 1993). Eight wells were used to make sure that sufficient assay precision is achieved and each of them was inoculated with 100 μl dilution. The viral CPE of the cells were observed daily for 5 days. For the TCID50/mL assessment, those highest dilutions were used in which 50% or higher wells were found positive.

### Statistical analysis

The experiments were carried out in multiples of three and were repeated at three different times, and the data are presented as the means with standard error. A *t*-test was performed to determine the significance using Microsoft Excel 2020.

## Results

### Minimum inhibitory and bactericidal concentrations

**Figure 1.**
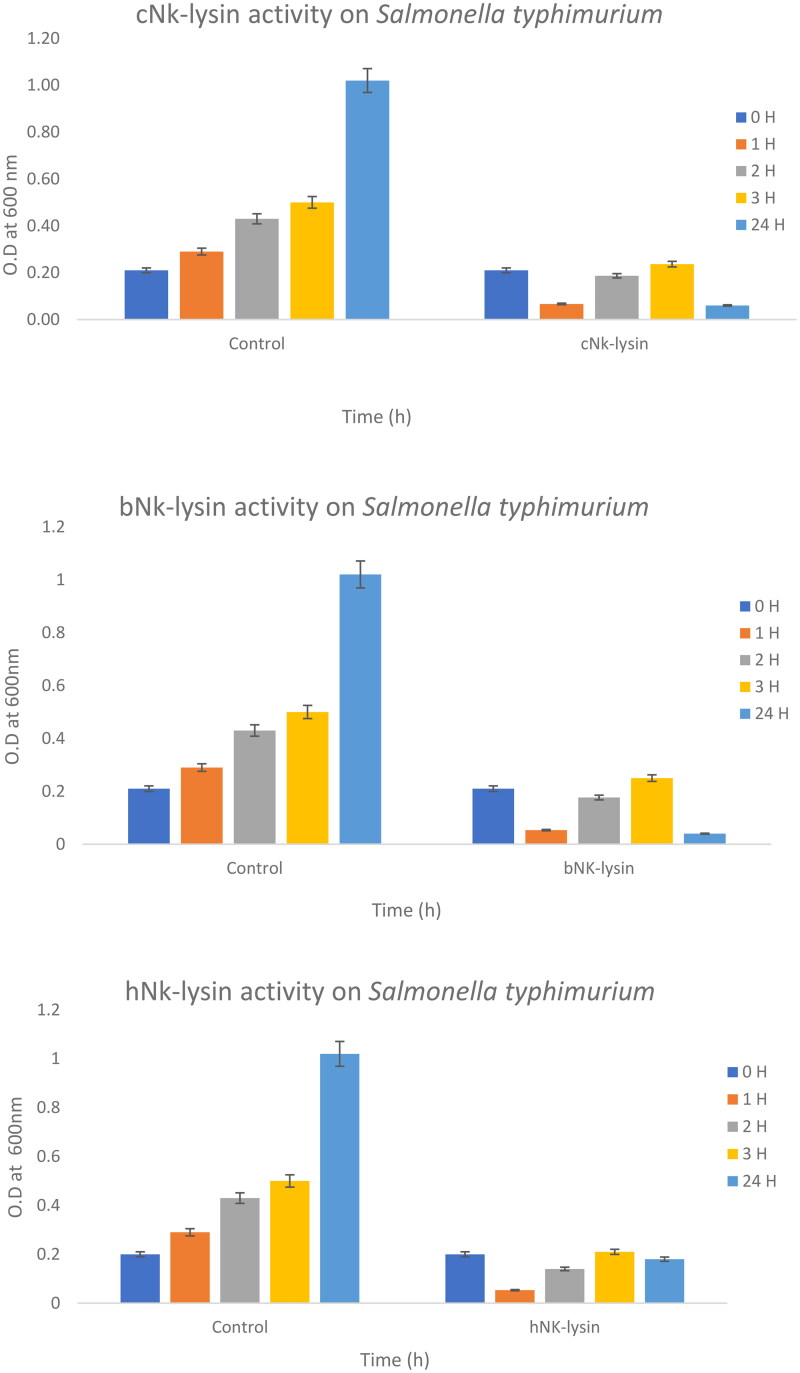
Antimicrobial activity of Nk-lysin peptides of chicken, bovine and human against *Salmonella typhimurium.* Data presented as means (±SD) of three independent repeats in triplicate (*p˂0.03 compared to control group).

This Example establishes the MIC and MCB, and further demonstrates the relative antibacterial activity of three different NK-lysin peptides from human, chicken and bovine activity directed against gram-negative and gram-positive bacteria. Figure 1 illustrates that all three NK-lysin peptides showed strong antibacterial activity against *Salmonella typhimurium* at a concentration of 15 μg/ml at three hours. The human NK-lysin peptide exhibited stronger antibacterial potential than peptides from chicken and bovine species after second and third hours of exposure and produced more than 60 and 50% reduction of bacterial survival at time intervals assayed. NK-lysin peptides of bovine and chicken exhibited strong antimicrobial activity (15 μg/ml) with less than 5% survival rate overnight, while human peptide displayed (82% killing) overnight.

The same assay was performed against additional bacterial strain, including *Streptococcus mutans*, *E. coli*, *Klebsiella oxytoca*, *Pseudomonas aeruginosa*, *Klebsiella pneumonia*, *S. sonnei* and *Streptococcus pyogenes*. Results are also summarized in Table 3, above. As shown, the NK-lysin peptides exhibited a wide range of activity against both gram-positive and gram-negative pathogens. The MIC and MBC values of chicken NK-lysin peptide ranged from 15 to 30 μg/ml displayed the maximal activity against all bacteria tested in the assays. However, similar results were recorded for bovine and human NK-lysin peptides, showing maximal activity of MIC at 15 μg/ml and MBC at 15–70 μg/ml, respectively. Notably, all NK-lysin peptides were found to be more efficient regarding bacterial inactivation as compared to the reference antibiotic, kanamycin, except in the case of *Shigella sonnei*, wherein kanamycin was shown to be more potent than the NK-lysin peptides.

**Table 3.** Antimicrobial activity of Nk-lysin peptides of chicken, bovine and human against gram-positive and gram-negative bacteria.

Bacterial strain	Nk-lysin chicken		Nk-lysin bovine		Nk-lysin human		Kanamycin	
	MIC (µg/ml)	MBC (µg/ml)	MIC (µg/ml)	MBC (µg/ml)	MIC (µg/ml)	MBC (µg/ml)	MIC (µg/ml)	MBC (µg/ml)
*Salmonella typhimurium ATCC 14028*	15 ± 0.098	30 ± 0.10	15 ± 0.10	25 ± 0.12	15 ± 0.090	70 ± 0.092	62.5 ± 0.00	62.5 ± 0.00
*Klebsiella Pneumonia (isolate)*	15 ± 0.030	25 ± 0.010	15 ± 0.03	25 ± 0.010	15 ± 0.040	15 ± 0.040	31.25 ± 0.00	31.25 ± 0.00
*St. pyogenes ATCC 19615*	15 ± 0.00	25 ± 0.010	15 ± 0.00	25 ± 0.010	15 ± 0.00	25 ± 0.010	62.5 ± 0.00	62.5 ± 0.00
*Streptococcus mutans (isolate)*	15 ± 0.00	15 ± 0.00	15 ± 0.0	15 ± 0.0	15 ± 0.010	25 ± 0.012	62.5 ± 0.023	62.5 ± 0.23
*Escherichia coli ATCC 11775*	15 ± 0.012	25 ± 0.010	15 ± 0.012	25 ± 0.010	15 ± 0.014	25 ± 0.012	15.62 ± 0.011	15.62 ± 0.00
*Klebsiella oxytoca ATCC 49131*	15 ± 0.015	25 ± 0.10	15 ± 0.015	25 ± 0.10	15 ± 0.012	15 ± 0.012	31.25 ± 0.00	31.25 ± 0.00
*Pseudomonas aeruginosa P.aeruginosa ATCC 9027*	15 ± 0.012	25 ± 0.013	15 ± 0.012	25 ± 0.013	15 ± 0.010	15 ± 0.010	62.5 ± 0.00	62.5 ± 0.00
*Shigella sonnei ATCC 25931*	15 ± 0.033	25 ± 0.010	15 ± 0.033	25 ± 0.010	15 ± 0.025	25 ± 0.012	7.81 ± 0.026	7.81 ± 0.026

Results of these assays showed that killing of bacteria by the NK-lysin peptides followed a dose-dependent pattern. Bacterial survival was less than 50% with treatment of each of the three peptides at low concentration (0.350 μg/ml). Chicken and bovine NK-lysin peptides displayed stronger antibacterial effects than human NK-lysin peptides under these test conditions. Accordingly, all three peptides demonstrated efficient antibacterial activities across a wide range of peptide concentrations.

### Colony count assays

**Figure 2.**
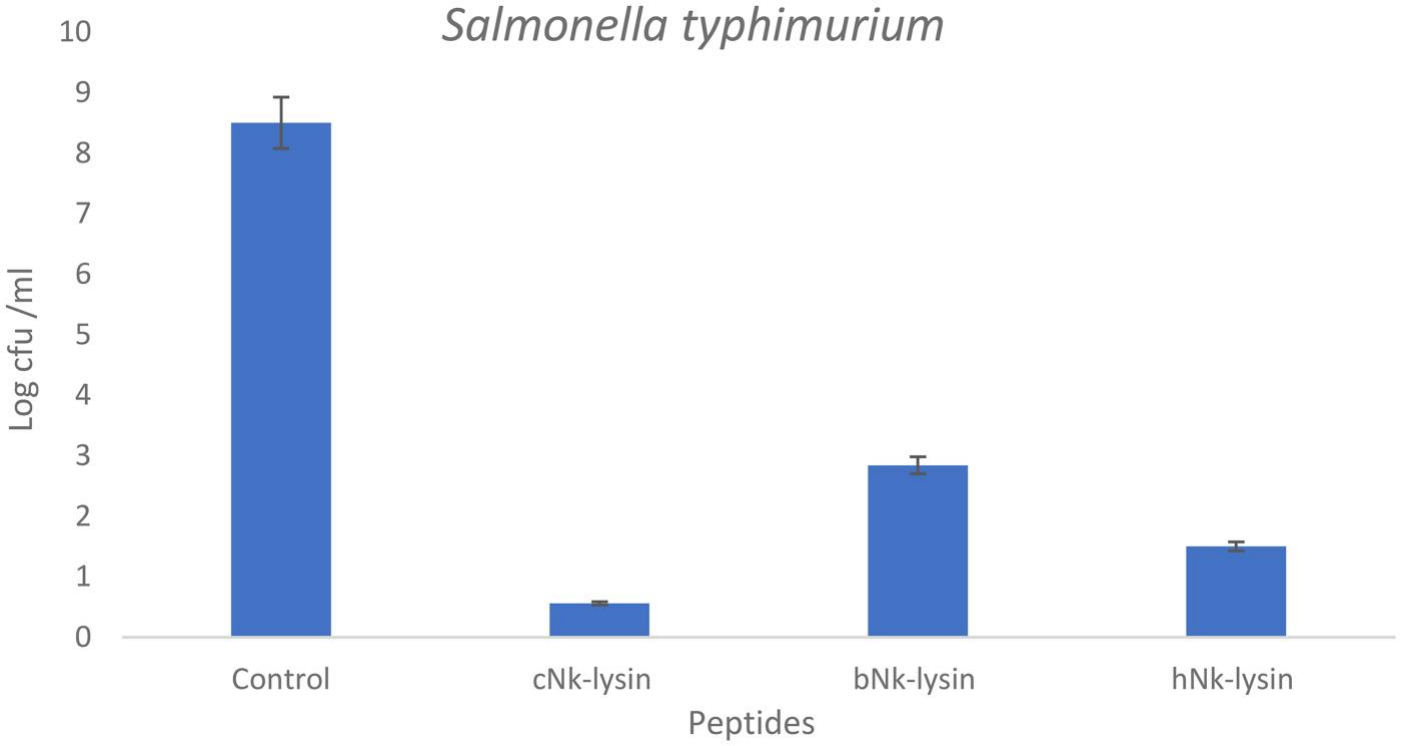
Colony counting assay of Nk-lysin peptides of chicken, bovine, human against *Salmonella typhimurium.* Data presented as means (±SD) of three independent repeats in triplicate (*p˂0.01 compared to control group).

The colony forming unit of *Salmonella typhimurium* was determined using NK-lysin peptides. Aliquots of *S. typhimurium* treated with the NK-lysin peptides were plated and counted after incubation in the presence of the peptides. Colony counts for an exemplary concentration of peptides are shown in (Figure 2) for *Salmonella typhimurium* treated with chicken, bovine or human NK-lysin to establish a value for colony forming units for each. For example, at 30 μg/ml concentration, chicken NK-lysin decreased the colony number by approximately 10-fold. Bovine NK-lysin at 25 μg/ml concentration around 8.6-fold decline in bacterial survival. A higher concentration of human NK-lysin peptide (70 μg/ml) led to decrease bacteria growth by 4.6-fold.

### Growth kinetic activity

**Figure 3.**
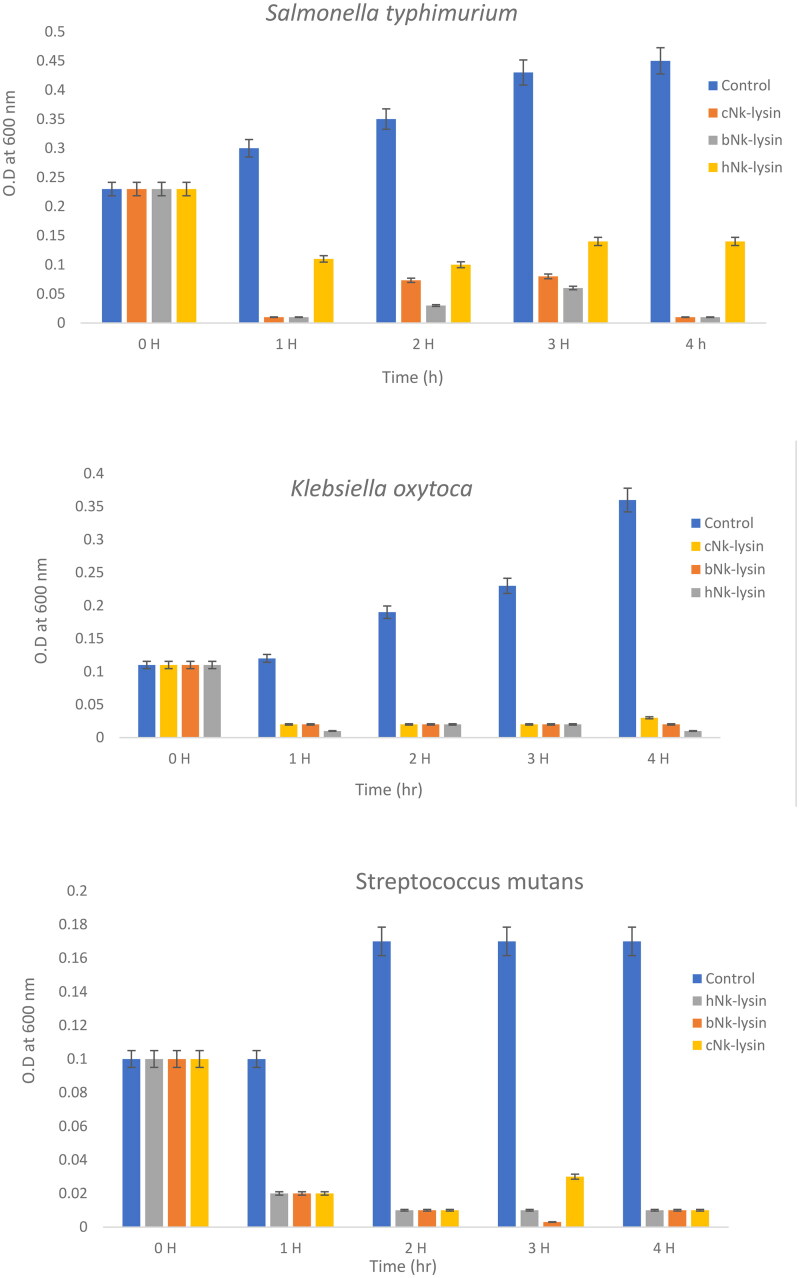

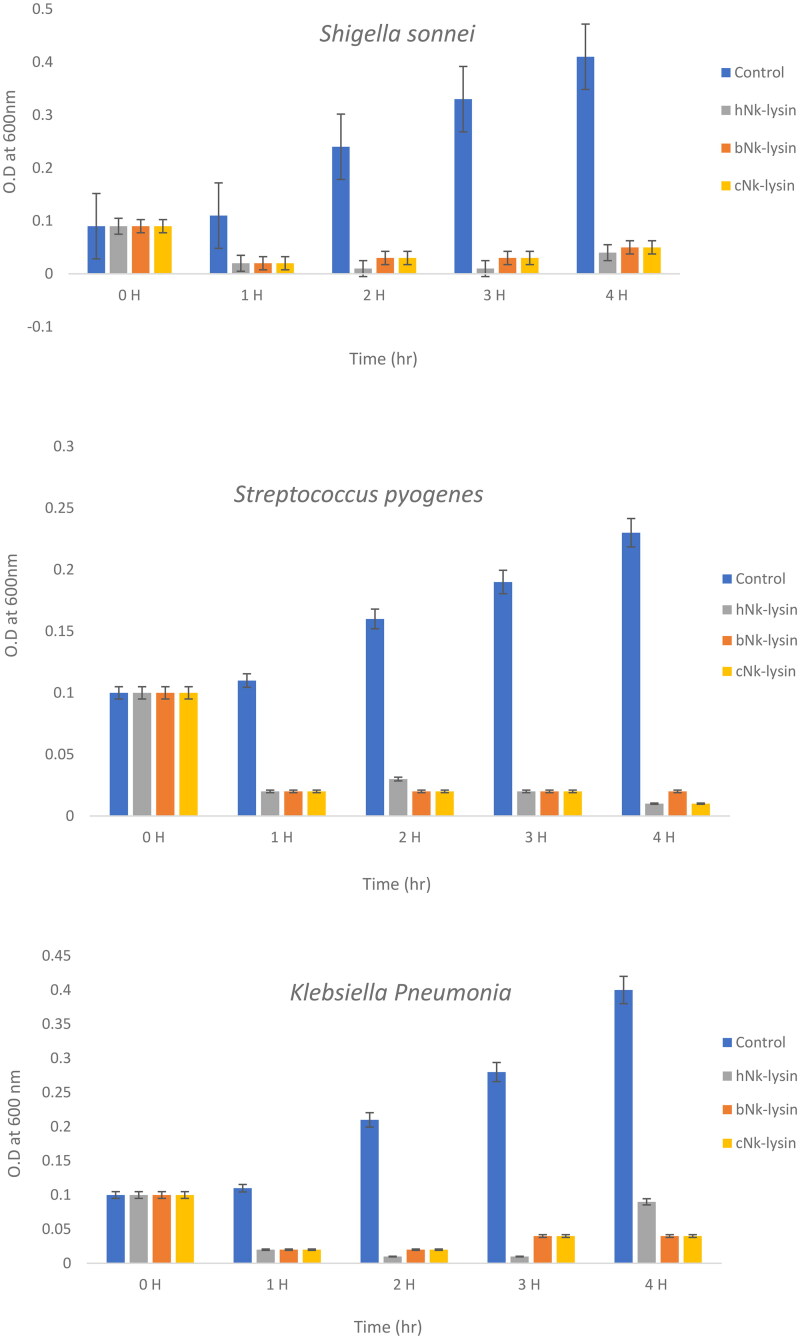

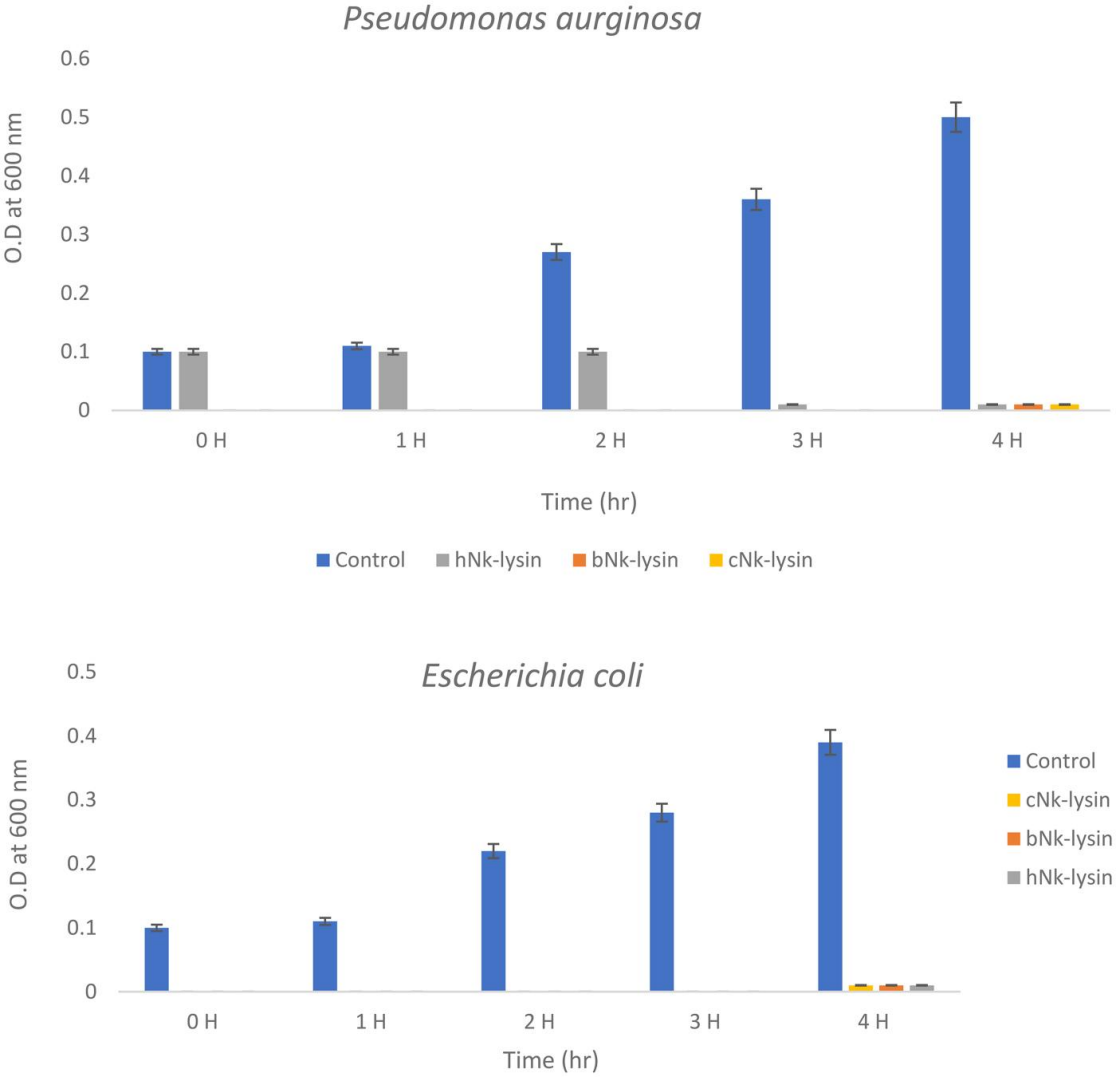
Growth kinetic activities of 2xMIC of chicken, bovine and human Nk-lysin peptide against *Streptococcus pyogenes* (ATCC 19615) (*p˂0.02 compared to control group) and *Streptococcus mutans* (isolate) (*p˂0.02 compared to control group), *Escherichia coli* (ATCC 11775) (*p˂0.03 compared to control group), *Klebsiella oxytoca* (ATCC 49131) (*p˂0.03 compared to control group), *Pseudomonas aeruginosa* (ATCC 9027) (*p˂0.04 compared to control group), *Salmonella typhimurium* (ATCC 14028), *Klebsiella Pneumonia* (isolate) (*p˂0.02 compared to control group)and *Shigella sonnei* (ATCC 25931) (*p˂0.03 compared to control group). Data presented as means (±SD) of three independent repeats in triplicate.

To observe time-dependent changes in the bacterial growth, the bacterial concentration and growth inhibition rates were further evaluated by measuring the OD at 600 nm at different time points shown in (Figure 3). The untreated bacterial growth curve reached the exponential phase rapidly and then followed a regular pattern comprising of a period of lag, an exponential phase followed by a stabilization phase. The growth of *Salmonella typhimurium* was decreased in lag phase at the first one hour, then enter exponential phase at the second hour with less than 20% survival rate for chicken and bovine NK-lysin peptides. The same scenario was detected at the third hour for both peptides. Complete inhibition was achieved after 4 hr of incubation at 2 × MIC of chicken and bovine NK-lysin.

For human NK-lysin peptide first hour, the growth of *Salmonella typhimurium* declined to 30% at lag phase, then was less than 40% when read at the 2 and 3 hr time points. At a concentration of 2 × MIC, the bacterial growth was less than 25% after incubation for 4 hr. Thus, bacterial growth inhibition not only depended on the peptide type but also on peptide concentration. In the case of *Klebsiella oxytoca*, complete inhibition of growth was achieved after 4 hr of incubation with the three NK-lysin peptides. This held true for *Streptococcus mutans* also, in that the dead phase occurred dramatically after exposure of NK-lysin peptides; however, the chicken NK-lysin peptide demonstrated more potent activity than bovine or human NK-lysin peptides, based on the kinetic activity and greatest level of efficacy in a time dependent manner regarding MIC or MBC. Kinetic growth activity was completely inhibited by incubation with NK-lysin peptides for *E. coli, Klebsiella aeruginosa*, *Klebsiella pneumoniae, Shigella sonnei* and *Streptococcus pyogenes* by the 3 hr readings.

### Bacterial lytic potential

**Figure 4.**
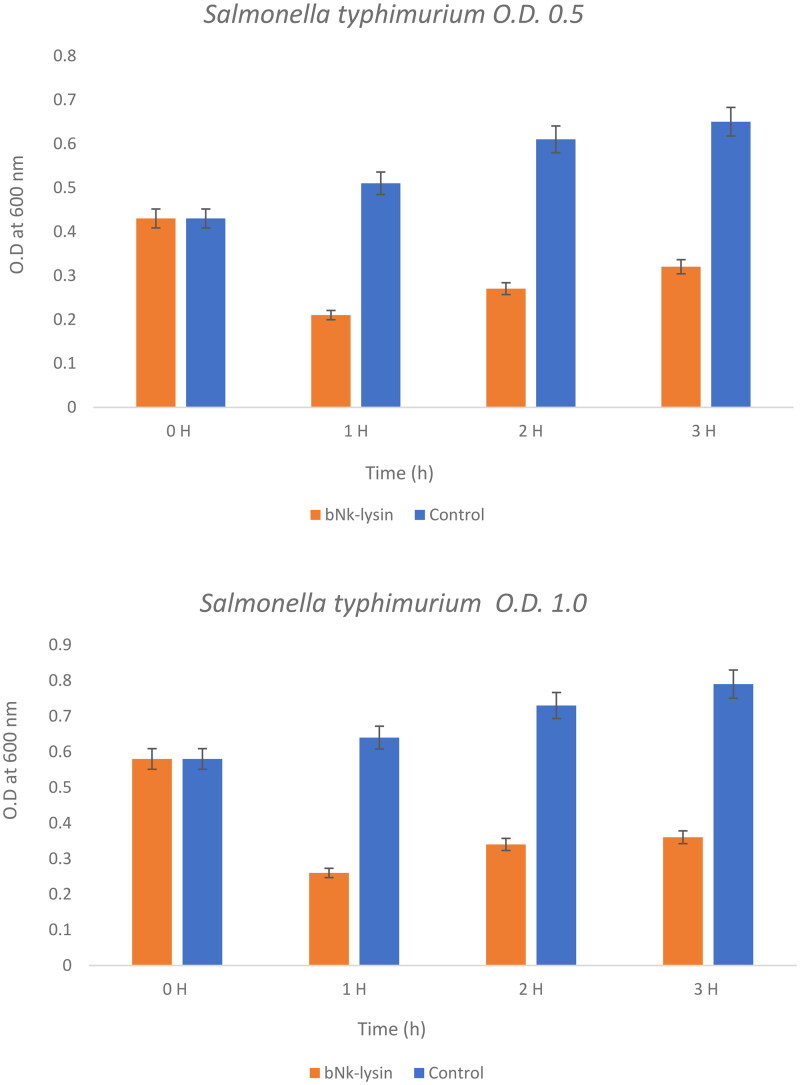
The bacterial lytic effect of 1× MIC bovine Nk-lysin peptide against Salmonella typhimurium (ATCC 14028) at different inoculum concentrations. Data presented as means (±SD) of three independent repeats in triplicate. (* p ˂ 0.01 compared to control group).

**Figure 5.**
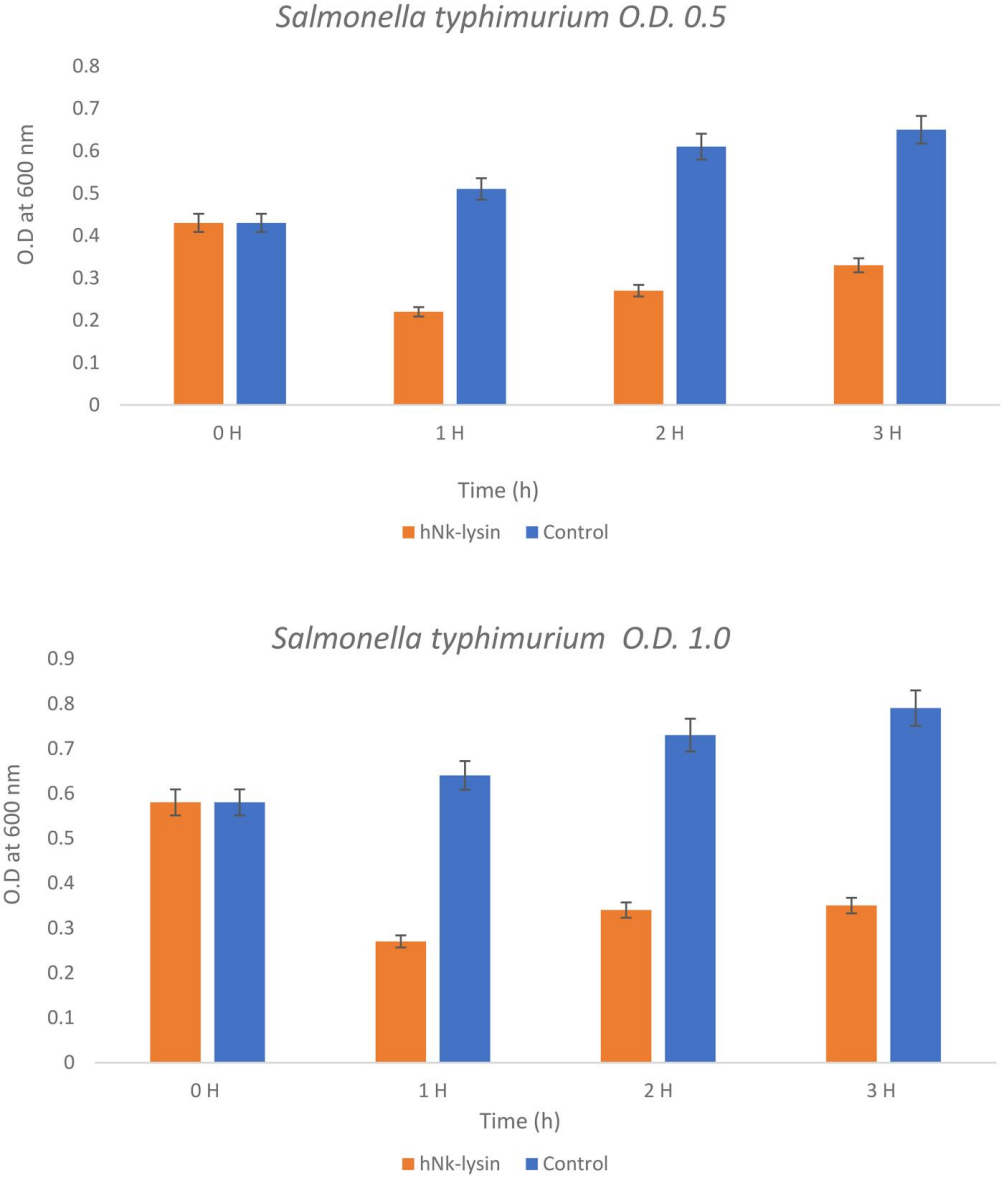
The bacterial lytic effect of 1x MIC bovine Nk-lysin peptide against *Salmonella typhimurium* (ATCC 14028) at different inoculum concentrations. Data presented as means (±SD) of three independent repeats in triplicate. (*p˂0.01 compared to control group).

This experiment poses the question as to whether these peptides would work at higher concentration of bacterial strains or not and compares the effect of bovine and human against two concentrations of *Salmonella typhimurium* at concentrations of (0.5) OD600 and (0.1) OD600) as seen (Figures 4 and 5). Bacteriolytic potential of bovine and human NK-lysin peptides were tested using two different inoculums of bacteria which were the OD600 (0.5) and OD600 (1.0) were used for screening. The bacterial inoculum of *Salmonella typhimurium* with concentration OD600 (0.5) incubated for 1 hr with bovine NK-lysin peptide had a survival rate that fell to approximately half of the starting culture, while the survival/growth of the control culture continued to survive and grow. After 2 hr of incubation bacteria survival was less than half of that measured for control. This trend continued even after 3 hr with onset of minor modification in growth of cell as depicted in (Figure 4) for bovine NK-lysin peptides.

The bacterial survivability in the presence of human NK-lysin peptides measured at OD600 (1.0) was 27, 34 and 35% after 1, 2 and 3 hours, respectively, passed. A similar trend was observed for bovine NK-lysin peptide, which is shown in (Figure 5), there is very little deviation. By incubating with 1 × MIC of peptide the lysis of cell was 79, 72 and 68% shown after 1, 2 and 3 hours have passed on the concentration of OD600 (0.5). Similar to human NK lysin peptide, at OD 600 (1.0) there was significant decrease in growth by 74, 76 and 64% after 1, 2 and 3 hours passed respectively. The lysis activity of both bovine and human NK lysin peptides at their respective MICs was similar as shown in (Figures 4 and 5).

### Mode of action of NK-lysin peptides

#### Leakage of intracellular contents

**Figure 6.**
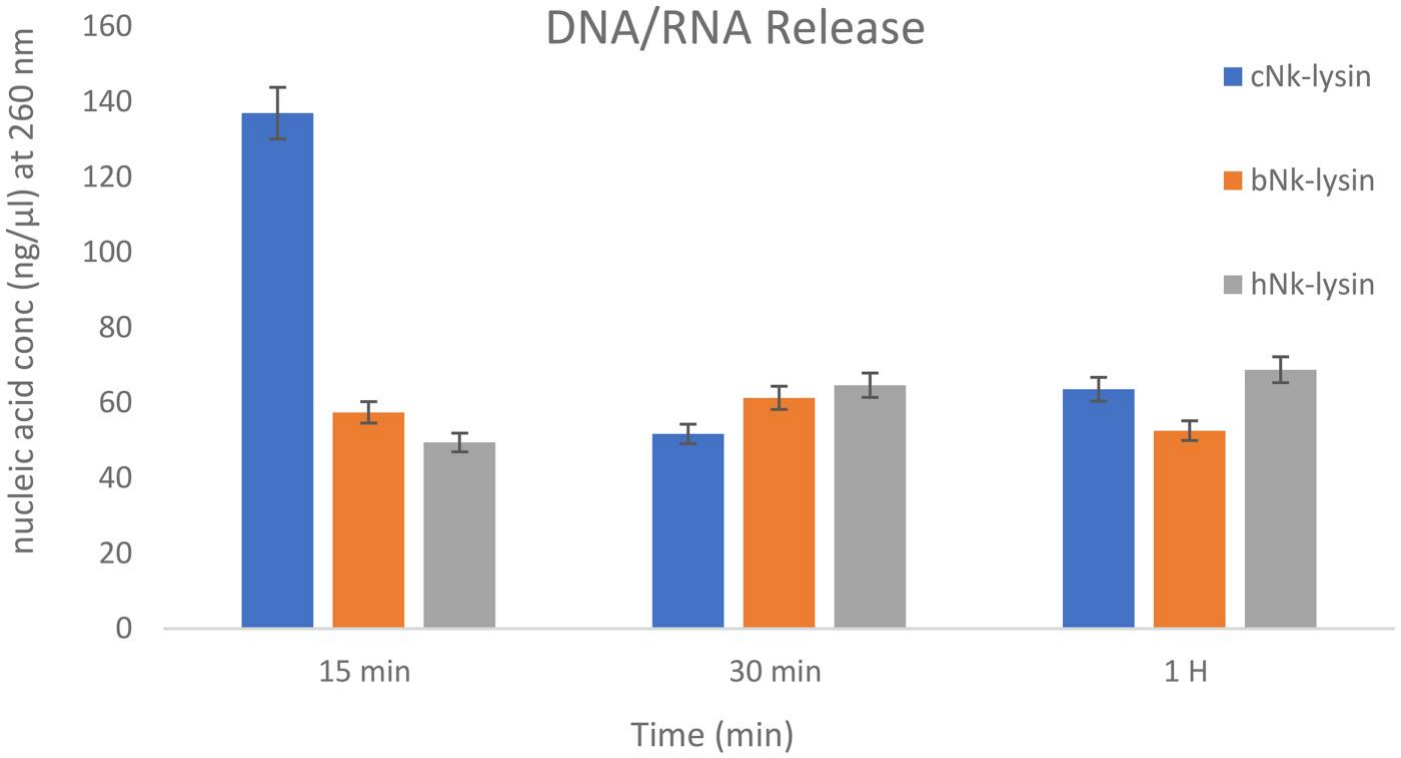
DNA/RNA release of *Salmonella typhimurium* (ATCC 14028) bacterial cells after incubation with (Nk-lysin peptides of chicken, bovine, human) at 37 °C. Data presented as means (±SD) of three independent repeats in triplicate. (*p˂0.01 compared to control group).

Inhibition of bacterial growth was thought to be due to membrane damage that occurs when the cultures are treated with NK-lysin peptides as depicted in (Figure 6). To confirm membrane damage, Nanodrop was used to quantify the release of nucleic acids from *S. typhimurium* after treatment with NK lysin peptide. The release of nucleic acids from *S. typhimurium* after membrane damage due to exposure to NK lysin peptide can be detected by reading changes in OD at 260 nm over time. After incubation periods of 15, 30 and 90 mins, OD 260 nm readings were taken. The chicken NK lysin peptide showed a higher activity (greater release of DNA/RNA) compared to the bovine and human NK-lysin peptides at 15 min, but there were no significant differences between the three peptides at 30 and 90 min, respectively. When NK lysin peptide isolated from humans were used the action of the peptide against *S. typhi* showed increased action at 15 min, compared to increase in action of chicken NK lysin at 30 and 90 min of exposure. These results were confirmed by gel electrophoresis and quantification a 500 bp fragment of 16s rRNA amplified using PCR. Amplified fragments were visualized with ethidium bromide staining and compared with markers of specific DNA ladder.

### Detection of resistance to beta-lactam and quinolone genes

**Figure 7.**
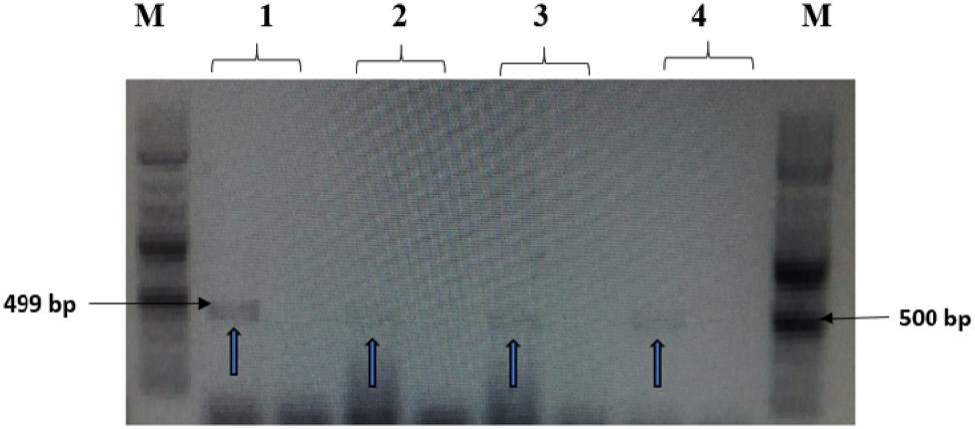
The amplified fragment of CTX-M1 gene with 499 bp, where M (DNA ladder =100 bp), 1 (*Salmonella typhimurium* ATCC 14028 untreated and treated cells), 2 (*Pseudomonas aeruginosa* ATCC 9027 untreated and treated cells), 3 (*Klebsiella oxytoca* ATCC 49131 untreated and treated cells), 4 (*Streptococcus pyrogens* ATCC 19615 untreated and treated cells) and arrows indicate gene in untreated bacteria whereas empty well indicates treated bacteria after challenge with Nk-lysin.

**Figure 8.**
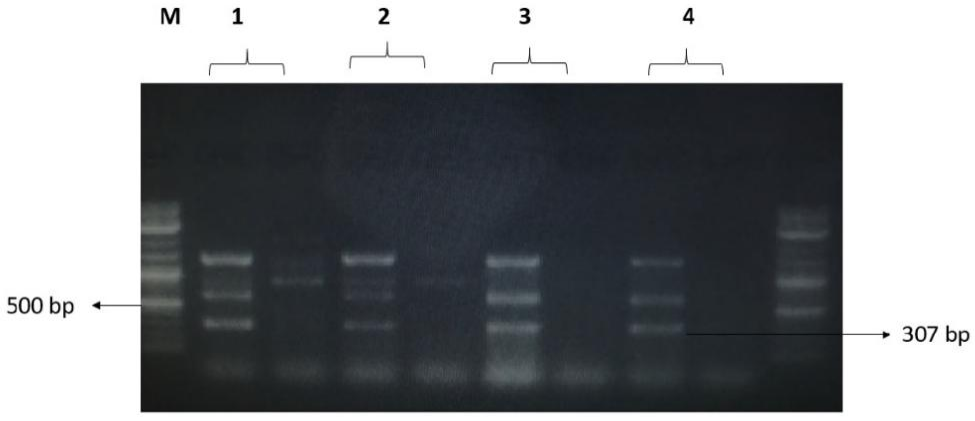
The amplified fragment of CTX-M8 gene with 307 bp, where M (DNA ladder =100 bp), 1 (*Salmonella typhimurium* ATCC 14028 untreated and treated cells), 2 (*Pseudomonas aeruginosa* ATCC 9027 untreated and treated cells), 3 (*Klebsiella oxytoca* ATCC 49131 untreated and treated cells), 4 (*Streptococcus pyrogens* ATCC 19615 untreated and treated cells) and arrows indicate gene in untreated bacteria whereas empty well indicates treated bacteria after challenge with Nk-lysin.

**Figure 9.**
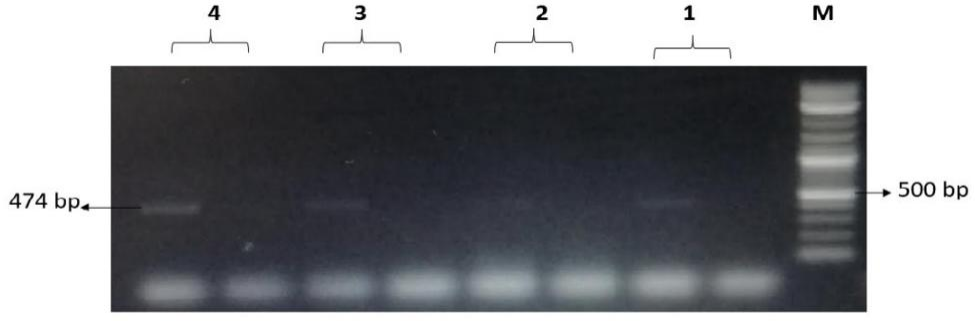
The amplified fragment of CTX-M9 gene with 474 bp, where M (DNA ladder =100 bp), 1 (*Salmonella typhimurium* ATCC 14028 untreated and treated cells), 2 (*Pseudomonas aeruginosa* ATCC 9027 untreated and treated cells), 3 (*Klebsiella oxytoca* ATCC 49131 untreated and treated cells), 4 (*Streptococcus pyrogens* ATCC 19615 untreated and treated cells) and arrows indicate gene in untreated bacteria whereas empty well indicates treated bacteria after challenge with Nk-lysin.

The treatments with NK-lysin peptide provided evidence of the presence/absence of plasmid-mediated quinolone-resistance and β-lactam-resistance genes for all gram negative and positive bacteria studies, as shown in Table 4. The dominant β-lactamase gene known as ^bla^TEM-1 was present in both treated and untreated bacteria, whereas Toho-1 was not found in untreated or treated bacteria. While β-lactamase genes CTX-M-1, CTX-M-8 and CTX-M-9 were detected in untreated bacteria, none of these were found in any of the bacterial stains after treatment with NK-lysin peptide, as shown in Figures 7–9. The presence of gyrase A, a chromosomal gene associated with topoisomerase 2, was detected in untreated and treated bacteria. In contrast, the QnrA, QnrB, QnrD and QnrS genes associated with plasmid-mediated quinolone-resistance were not detected in any bacteria, irrespective of whether untreated or treated. These data indicate that NK-lysin peptide treatment effectively inhibited β-lactamase genes ^bla^TEM-1, CTX-M-1, CTX-M-8 and CTX-M-9 were inhibited or suppressed and may be used in combination with one or more (3-lactam antibiotics to treat an infection caused by β-lactam-resistant bacteria.

**Table 4.** Detection of beta-lactam and quinolone resistance genes after treatment with Nk-lysin peptides.

Gene name	Untreated bacteria	treated bacteria
BLATem	(+) all	(+) all
TOHO1	(−) all	(−) all
CTX-M1	(+) all	(−) all
CTXM8	(+) all	(−) all
CTXM9	(+) all	(−) all
Gyrase A	(+) all	(+) all
QnrA	(−) all	(−) all
QnrB	(−) all	(−) all
QnrD	(−) all	(−) all
QnrS	(−) all	(−) all

(+) all, the gene is present in all bacteria.

(−) all, the gene is absent in all bacteria.

### Cytotoxicity and antiviral activity of Nk-lysin peptides

Rotavirus (strain SA-11) was used to evaluate the antiviral action of the three NK-lysin peptides in a mammalian cell type *in vitro*. MA-104 cells were first tested to confirm that the peptides alone were safe and nontoxic. Cytotoxity of the NK lysin peptides at a concentration of 80 μg/ml was then tested against rotavirus infection of MA-104 cells. Each species of the NK-lysin peptides was combined with an equal volume of the rotavirus stock solution described above and incubated for 1 hr at 37 °C. Microplates with MA-104 cells plated at equal density were preincubated and then treated with an array of rotavirus/peptides mixtures except for wells that were untreated control MA-104 cells. The microplates were incubated for 5 days at 37 °C. under 5% CO_2_. Data is summarized in Table 5 showing various levels of antiviral activity against the rotavirus. The bovine NK-lysin peptides showed the highest degree of antiviral activity, with 90% inhibition of infection. Chicken NK-lysin peptide showed similar results with 80% inhibition, whereas inhibition with human NK-lysin peptide was the lowest at 50% inhibition.

**Table 5.** The antiviral activity of Nk-lysin peptides of chicken, bovine and human at concentration (80 µg/ml) against Rotavirus sa-11.

Tested extract	TCID50/ml	Log_10_TCID50/ml	Log reduction	% Reduction
Initial titer	1.95 × 10^6^	6.29	N.A	N.A
cNk-lysin	4.11 × 10^5^	5.61	0.7	79%
bNk-lysin	1.95 × 10^5^	5.29	1.00	90%
hNk-lysin	1.00 × 10^6^	6.00	0.29	49%

## Discussion

The antibacterial, antiviral activities and mechanism of action of NK-lysin peptides are explained in detail in the examples that follow. The results revealed that the NK lysin peptide inhibited bacterial activity very well, especially in the first hour, when the inhibition rate was 95%, which was notably true for *Salmonella typhimurium*. The results demonstrate that the NK lysin peptide has a high level of efficacy against all gram-negative and gram-positive bacteria tested.

Synthetic NK lysin peptides (functional region helices 2 and 3) showed similar hydrophobicity (40–43%), net positive charge (5.0–7.9), and basic residues of (20–30%) which led to high effectiveness against *S. aureus* and *E. coli*.^33^ The high alpha helicity of the bovine peptide did not exhibit much effect on *M. bovis* and *M. hemolytic* isolates,^33^ however it was reported to be highly effective against *H. Somni* isolates. Several studies reported similar antimicrobial activity by chicken NK lysin peptide compared to other NK lysin peptides.^29^^,^^34^^,^^35^

In an earlier study^36^ observed rapid permeability of *Trypanosoma cruzi* plasma membrane resulting in the release of cytosolic enzymes within minutes of exposure to mammalian Nk-lysin peptide. They also found that the NK-lysin and NK-2 killed the trypanosomes residing inside the human glioblastoma cells (86HG39), but only NK-2 left the host cells apparently unharmed whereas Nk lysin was found to be harmful.

Our results demonstrated that when compared to kanamycin, NK-lysin peptides demonstrated greater antibacterial effectiveness. However, the MIC values for all NK-lysin peptides were determined to be 15 µg/ml for all bacterial strains examined. Kanamycin’s MIC values varied from 7.8 to 62.5 µg/ml. Kanamycin was effective at a dose of 62.5 µg/ml against *Salmonella typhimurium, Streptococcus pyogenes, Streptococcus mutans* and *Pseudomonas aeruginosa*, but only 31.25 µg/ml against *Klebsiella pneumonia* and *Klebsiella oxytoca. Shigella sonnei’s* MIC was determined to be 7.8 µg/ml of kanamycin.

A kinetic investigation of the three NK-lysin peptides revealed that the chicken and bovine NK-lysin peptides exhibited comparable levels of activity against *Salmonella typhimurium*, with significant mortality at 1 hour after treatment. Human NK-lysin peptides were less effective, with a 75% decrease in *Salmonella typhimurium* growth after 4 hours. Chicken NK-lysin peptides were far more effective, and other bacteria tested did not survive even 3 hours after treatment. The variations in kinetics that characterize the antibacterial effect of the peptide may be attributable to hydrophilic areas and positively charged amino acids that are mostly located on the surface area of these peptides, without being restricted by theoretical ideas. This may influence the mode of action of the majority of NK-lysins peptides.

The findings are consistent with prior research, which discovered that the intramolecular structures of NK-lysin are linked by six cysteine residues that form a bridge between helical 1/helical 4/helical 5 units and helical 2/helical 3 units. The helical peptide contains a Trp residue, which has been proven to contribute to a greater rate of insertion into bacterial membranes.^37^ Pheasant cathelicidin-1 (Pc-CATH1) showed similar data, as the growth of *E. coli* was restricted after 1 hour of exposure, and the bacteria did not start growing even after 6 hours of exposure.^38^

Melimine peptide and its derivative exhibited a powerful antimicrobial activity against *P. aeruginosa* because their smaller size (17–29 amino acid), which provided the cytoplasmic membrane covering capability in *P. aeruginosa.*^28^ It takes more time to move across the outer membrane or interact with the inner membrane of *P. aeruginosa* to destroy the bacteria, or it needs to align itself inside the membrane more effectively to start its activity.

These NK-lysin peptides used a bacterial lytic potential against a high inoculum of *S. typhi*, where there was 80% decline in bacterial growth following 1 × MIC incubation. Their small size (30 amino acids) and structure led to this potent activity. This finding was coherent with other studies that showed that the 15–20 amino acid residues is required for the peptides to cover bacterial cytoplasmic membranes.^39–41^

The quantity of non-polar amino acids at the side chain of NK-lysin peptides plays an important role in moving across the bacterial membrane, since these peptides include 27–51% of non-polar amino acids across all animal species.^42^ Non-polar amino acids, such as Ile and Leu, are known to enhance peptide binding and cell membrane rupture. NK-lysin peptides are saposin family members with modest differences in domain length. According to species, the domain is folded into 4–5 helical bundles with three disulfide bonds between six cysteine residues that stabilize the peptide conformational shape.^43^

These findings are consistent with previous research that found five helical folded structures in a single globular chain of the peptide and stable disulfide bridges between six cys residues in porcine NK-lysin peptide, whereas the human NK-lysin domain contains four cysteine residues that form two disulfide bonds. The NK-lysin peptides’ small size (30 amino acids) and structure may also contribute to their potent activity, as studies of other antimicrobial peptides have suggested that an optimal amino acid length for the peptides to cover bacterial cytoplasmic membranes should be around 15–20 residues.^15^^,^^42^^,^^44^

Within 15 minutes of incubation, the three different NK-lysin peptides caused a concentration-dependent release of DNA/RNA (260 nm absorbing material). This was in consistence with the earlier findings that Enterocin CRL35, melimine and its derivative Mel4 stimulate the release of DNA/RNA from *Listeria monocytogenes* and *Pseudomonas aeruginosa* in a concentration-dependent manner.^28^^,^^45^ There was no significant difference in the release of DNA/RNA after 90 minutes of exposure to chicken, bovine or human NK-lysin peptides.

Because the conformational structure of NK-lysin and granulysin peptides is homologous, they appear to act through the same pathway leading to bacterial inactivation, which is mediated by physical interactions between positively charged peptides and negatively charged cell membrane bilayer phospholipids, resulting in membrane disruption.^42^^,^^46^ Without being bound by theory regarding this mode of action, DNA/RNA is released as a result of nucleic acid bursting and disintegration, which may occur during bacterial apoptosis-like death that is similar to eukaryotic cells that caused physiological and biothermal variations following peptide exposure.^15^ NK-2 similarly dissolved parasitic membranes to release a cytosolic marker protein, suggesting that both mammalian NK-lysin and NK-2 target the parasite’s plasma membrane.^47^

Among the topics that have been studied most often in antibiotic resistance field is β-lactam resistance with specific emphasis on β-lactamases, the enzymes that are capable of hydrolyzing β-lactam antibiotics.^48^ Since the 1980s, the number of β-lactamases has increased significantly; especially class A and D, β-lactamases. From the class A β-lactamases, extended-spectrum β-lactamases (ESBLs) that are able to hydrolyze expanded spectrum cephalosporins (cefotaxime, ceftriaxone, ceftazidime or cefepime) and monobactams (aztreonam) are cause of a major public health concern.^49^

Class A ESBLs essentially comprise of TEM, CTX-M, SHV, GES and VEB enzymes, out of which the greatest number of variants defined in the previous years are part of the CTX-M family (total of 180 variants).^50^ In our study, the presence of the dominant TEM-1 gene was found in all untreated and treated bacteria, however the TOHO-1 gene was not found in any of them. Importantly, the β-lactamase genes CTX-M-1, CTX-M-8 and CTX-M-9 were discovered in untreated bacterial strains but not in any bacterial strains following treatment with NK-lysin peptides. Thus, our findings provided a unique therapy by inhibiting synthesis of β-lactamase genes in β-lactam antibiotic-resistant bacterial infections that used NK-lysin peptides.

Our results did not find any of the tested plasmid-mediated quinolone resistance genes either in the treated or untreated bacterial strains. We hypothesized that the absence of plasmid-mediated quinolone resistance genes and beta-lactam resistance genes is due to plasmid curing and down-regulation of beta-lactamase gene expression enzymes (CTX-M1, M-8 and M-9) in response to Nk-lysin peptide exposure. This manner of action for these peptides and other innate immune system components has never been documented.

A plasmid cured derivative is often sought with certain plasmid-containing bacteria so that a direct comparison can be performed between the two. Certain plasmids go through spontaneous segregation and deletion. But most of them are quite stable and need curing agents or other conditions (increased growth temperature, thymine starvation) to enhance the frequency of spontaneous segregation so that majority of the plasmids can enter the bacterial host chromosome. When this happens, the plasmid would no longer be present as a covalently closed circular (CCC) molecule.^51^

Consequently, NK-lysin treatment may also lead to the genetic re-organization of beta-lactamases enzymes during cell replication, which may cause a change in the location or integration of transposon elements. These enzymes are differentiated through β-lactamase (bla) genes in plasmids or chromosomes. However, this genetic feature is no longer used because it is possible to mobilize and integrate chromosomal bla genes into plasmids or transposons; however, cases of initially described plasmid-mediated β-lactamases entering into the chromosome have also been noted.^52^^,^^53^ Furthermore, protein regulation (constitutive or inducible expression) has also been noted with respect to various β-lactamase groups.^54^

Bla genes may be absent in certain isolates with decreased susceptibility in *Salmonella* possibly because of weak sensitivity of phenotypic resistance detection approaches, down-regulation of outer membrane porins,^55^ changes in the beta-lactam targets (PBPs),^56^ varied ampC beta lactamases,^57^ and overexpression of efflux resulting the decrease in susceptibility. There are certain genetic elements and plasmids with blaCTX-M genes that also include other resistance genes, such as those encoding AmpC β-lactamases (plasmid blaAmpC) and carbapenemases, methylases affecting aminoglycosides or plasmid-mediated quinolone resistance (PMQR) genes.^58^

These genes may also benefit blaCTX-M for maintenance because of the co-selection procedures;^50^ therefore, it may result loss of few CTX-M genes during cell division. IS located upstream, like ISEcp1, have demonstrated the experimental mobilization of blaCTX-Ms genes.^59^

Chicken, bovine and human NK-lysin peptides are demonstrated herein to have antibacterial activity and antiviral activity against Rotavirus (strain SA-11). On the basis of the comparison between these peptides, potent antiviral activity of bovine NK-lysin against Rotavirus (strain SA-11) is particularly evident, inhibiting infection by up to 90%. However, growth was also significantly inhibited by chicken and human NK-lysin peptides, restricted by 80 and 50%, respectively.

Though the studies on Nk-lysin peptides have been carried out in terms of their antibacterial activity, the potential antiviral activity of other innate immunity molecules, apart from Nk-lysin, was taken by us. For example, antiviral activity of the human cathelicidin LL-37 was observed against, IAV, adenovirus, HIV and respiratory syncytial virus.^60–62^ Furthermore, it has also been determined that defensins, such as α- and β-defensins, have antiviral activity against IAV.^63^

It was found in various studies that there are antiviral effects of innate immune response molecules on enveloped and non-enveloped viruses and without being bound by theory, it is assumed that membrane disruption is one of the antiviral methods exhibited by defensins against enveloped viruses.^59^

According to Falco et al.,^64^ NK-lysin short peptide of the turbot (*Scophthalmus maximus*) demonstrated powerful antiviral activity against spring viremia of carp virus (SVCV) not only by preventing viral particles from binding to host cells, but also by preventing the integration of virus and cell membranes that require a low pH. Other antiviral methods of these peptides appear to rely on either specific binding to specific viral proteins or nonspecific lectin-like binding to virus envelope glycoproteins. Taking this mechanism of action into account, there is evidence that defensins may have inhibitory effects by interfering with the fundamental interaction between the influenza glycoprotein hemagglutinin and the cellular receptor sialic acid.^66–70^

## Conclusion

Our research shown that NK-lysin was effective against a wide variety of bacteria and viruses, including gram-negative and gram-positive strains of bacteria and viruses, as well as a number of common food-borne pathogens. We found that the beta-lactamase genes (CTX-M-1, M-8 and M-9) were down-regulated when treated with NK-lysin peptide. The bacterial strains that were screened did not contain any of the plasmid-mediated quinolone resistance genes. Results showed that Nk-lysin peptides had potent antiviral effects against Rotavirus (strain SA-11), reducing infection by around 90% with chicken Nk-lysin, 80% with bovine Nk-lysin, and 50% with human Nk-lysin. There have been studies on NK-lysin peptides and their antibacterial activity against certain microbes, but whether NK-lysin peptides from bovines, humans or chickens can be used as antimicrobial and/or antiviral agents on their own or in combination with other agents is still up for discussion. Additionally, there is a continuous and pressing requirement for the possibility of making antibiotic-resistant bacterial infections susceptible to beta-lactam antibiotics.
